# Geographic mapping of choroidal thickness in myopic eyes using 1050-nm spectral domain optical coherence tomography

**DOI:** 10.1142/S1793545815500121

**Published:** 2015-07-01

**Authors:** Qinqin Zhang, Maureen Neitz, Jay Neitz, Ruikang K. Wang

**Affiliations:** * Department of Bioengineering, University of Washington, Seattle, WA 98195, USA; † Department of Ophthalmology, University of Washington, Seattle, WA 98104, USA

**Keywords:** Choroidal thickness, myopia, SDOCT, geographic mapping

## Abstract

**Purpose:**

To provide a geographical map of choroidal thickness (CT) around the macular region among subjects with low, moderate and high myopia.

**Methods:**

20 myopic subjects (*n* = 40 eyes) without other identified pathologies participated in this study: 20 eyes of ≤ 3 diopters (D) (low myopic), 10 eyes between −3 and −6D (moderate myopic), and 10 eyes of ≥ 6D (high myopic). The mean age of subjects was 30.2 years (± 7.6 years; range, 24 to 46 years). A 1050 nm spectral-domain optical coherence tomography (SD-OCT) system, operating at 120 kHz imaging rate, was used in this study to simultaneously capture 3D anatomical images of the choroid and measure intraocular length (IOL) in the subject. The 3D OCT images of the choroid were segmented into superior, inferior, nasal and temporal quadrants, from which the CT was measured, representing radial distance between the outer retinal pigment epithelium (RPE) layer and inner scleral border. Measurements were made within concentric regions centered at fovea centralis, extended to 5 mm away from fovea at 1 mm intervals in the nasal and temporal directions. The measured IOL was the distance from the anterior cornea surface to the RPE in alignment along the optical axis of the eye. Statistical analysis was performed to evaluate CT at each geographic region and observe the relationship between CT and the degree of myopia.

**Results:**

For low myopic eyes, the IOL was measured at 24.619 ± 0.016 mm. The CT (273.85 ± 49.01 µm) was greatest under fovea as is in the case of healthy eyes. Peripheral to the fovea, the mean CT decreased rapidly along the nasal direction, reaching a minimum of 180.65 ± 58.25 µm at 5 mm away from the fovea. There was less of a change in thickness from the fovea in the temporal direction reaching a minimum of 234.25 ± 42.27 µm. In contrast to the low myopic eyes, for moderate and high myopic eyes, CTs were thickest in temporal region (where CT = 194.94 ± 27.28 and 163 ± 34.89 µm, respectively). Like the low myopic eyes, moderate and high myopic eyes had thinnest CTs in the nasal region (where CT = 100.84 ± 16.75 and 86.64 ± 42.6 µm, respectively). High myopic eyes had the longest mean IOL (25.983 ± 0.021 mm), while the IOL of moderate myopia was 25.413 ± 0.022 mm (**p < 0.001). The CT reduction rate was calculated at 31.28 µm/D (diopter) from low to moderate myopia, whilst it is 13.49 µm/D from moderate to high myopia. The similar tendency was found for the IOL reduction rate in our study: 0.265 mm/D from low to moderate myopia, and 0.137 mm/D from moderate to high myopia.

**Conclusion:**

The CT decreases and the IOL increases gradually with the increase of myopic condition. The current results support the theory that choroidal abnormality may play an important role in the pathogenesis of myopic degeneration.

## 1. Introduction

Myopia, commonly known as nearsightedness, is an eye condition where light coming through the pupil does not directly focus on the retina, but in front of it. This makes the image of distant objects out of focus, while near objects remain clear. Myopia is one of the most common eye conditions and the leading cause of visual impairment, but the mechanism is still unknown. The proportion of people with myopia has been found as high as 80% in Chinese students.^1^ The proportion of patients with high myopia is reported to be 1–2% in the United States, 5–8% in Japan, 15% in Singapore, and 21–38% in Taiwanese students.^2,3^ Research suggests the prevalence has increased dramatically in recent decades. Myopia is measured in diopters (D) by the strength or optical power of a corrective lens that focuses distant images on the retina. It has also been classified by the degree of severity^4,5^: Low myopia usually describes myopia of −3.00D or less (i.e., closer to 0.00). Moderate myopia usually describes myopia between −3.00 and −6.00D. Those with moderate amounts of myopia are more likely to have pigment dispersion syndrome or pigmentary glaucoma.^6^ High myopia is defined as −6.00D or more.^5^ Roughly 30% of myopes have high myopia^7^ and people with this condition are more likely to have retinal detachments^8^ and primary open angle glaucoma.^9^ They are also more likely to experience floaters, shadow-like shapes which appear singly or in clusters in the field of vision.^10^

Myopic degeneration is a condition associated with the axial elongation of the globe, together with thinning and stretching of retina, retinal pigment epithelium (RPE) and choroid. Intraocular axial length (IOL) is currently the primary determinant of non-syndromic myopia, which defines the distance along the optical axis of the eye from the anterior curvature of the cornea to the retinal pigment. IOL is one of the basic anatomic parameters in ophthalmology and a major variable in determining the optical quality of the image on the retina. Currently, the IOL is usually measured by the IOL Master (Carl Zeiss Meditec Inc) with micro-scale resolution.

The choroid is a layer of vascular bed lying between the retina and the sclera, with a main function of providing oxygen and nourishment to the RPE cells and the outer layers of the retina. In addition, the choroid also contributes to the blood supply of the prelaminar portion of the optic nerve. Compromised choroidal circulation may account for retinal dysfunction and vision loss that is often observed in myopic and glaucomatous patients. Therefore, choroid thickness (CT) may be another important parameter for the investigations of vision loss. However, with the currently available optical techniques, it is difficult to image the full thickness of the choroid largely due to the pigment granules in the RPE layer that cause strong light scattering and absorption. In prior studies of CT in myopia,^11–18^ researchers have focused on measuring high myopia (≥ 6D). This is helpful for the study of retinal detachment^8^ and primary open angle glaucoma. However, in order to study the progression of myopia, measurement of CT should also be considered with the patients with low and moderate myopic conditions. Until recently, indocyanine green angiography was the best diagnostic method for diseases involving choroidal circulation and anatomy. It has good penetration, allowing visualization of choroidal vessels, but it does not provide the ability to quantify the CT.

Spectral domain optical coherence tomography (SD-OCT) is a noninvasive, noncontact, high-resolution, high-sensitive and depth-resolved optical imaging technology based on low-coherence spectral interferometry.^19^ Thanks to the recent advances in the development of light sources and linear detector arrays, SD-OCT has seen rapid adoption and wide spread use in ophthalmic imaging applications, including both clinical and research. The most popular light source employed in current ophthalmic OCT systems is a 840 nm wavelength-band light source. However, it is now known that the 840 nm light source is susceptible to strong scattering and absorption due to melanin in RPE, limiting its penetration into the deep choroid. In order to enhance light penetration into the choroid, a 1050 nm wavelength light source has been shown as a preferable choice for OCT implementation because it possesses lower absorption and scattering by the melanin when compared to that of 840 nm.^20–22^ There are reports that describe the successful use of 1050 nm SD-OCT system to provide quantitative measures of the CT.^23^ No reports so far, however, have investigated the use of 1050nm SD-OCT to provide the geographic maps of CT distribution among individuals with low, moderate and high myopia. Furthermore, here, CT and IOL were measured simultaneously, obviating the need for a separate procedure for the measurement of IOL in each subject. This could decrease health care cost and increase patient compliance for such procedures.

In this paper, we report a newly developed ultrahigh speed 1050nm SD-OCT system that is able to provide the geographic mapping of the choroidal thickness (CT) in patients with myopia. This newly developed system has some unique features compared to conventional systems, which are particularly useful in the practical imaging of myopic eyes in patients. These features include (1) 120 kHz line scan rate and (2) relatively long ranging distance of 6.1mm, while retaining the system resolution of < 10 µm. These features provide the operator with a better retinal orientation while positioning the target which is an important factor in decreasing patient examination time. More importantly the system is capable of simultaneously providing the measurement of IOL and the imaging of 3D choroid in one scan. With these improved features of the system, the aim of our study is to measure the macular CT and IOL and investigate the relationship between these parameters and degree of myopia.

## 2. Methods and Materials

### 2.1. Human subjects

A total of 20 myopic volunteers for a total of 40 eyes (age: 24–46, mean age: 30.2 ± 7.6) were recruited for the study. Exclusion criteria included history of proliferative retinopathy, epiretinal membrane, retinal detachment, retinal hemorrhage, choroidal neovascularization, macular hole, myopic macular atrophy, or amblyopia. Among the 40 eyes, 20 were categorized as low myopia with refractive errors of less than −3D. About 10 eyes fell between −3 and −6D and were categorized as having moderate myopia. The remaining 10 eyes suffered from high myopia (≥ −6D). The 1050 nm SD-OCT system (see below) was used to acquire 3D OCT images of the posterior segment approximately centered at fovea in the 40 eyes, upon which segmentation software was used to extract the geometric thickness of the choroid. During imaging, the OCT light power used at the eye pupil was ∼ 1.8 mW, which is within the safe ocular exposure limits recommended by the American National Standards Institute (ANSI).^24^

The protocol for this retrospective study was approved by the Institutional Review Board of University of Washington.

### 2.2. System setup

**Fig. 1:**
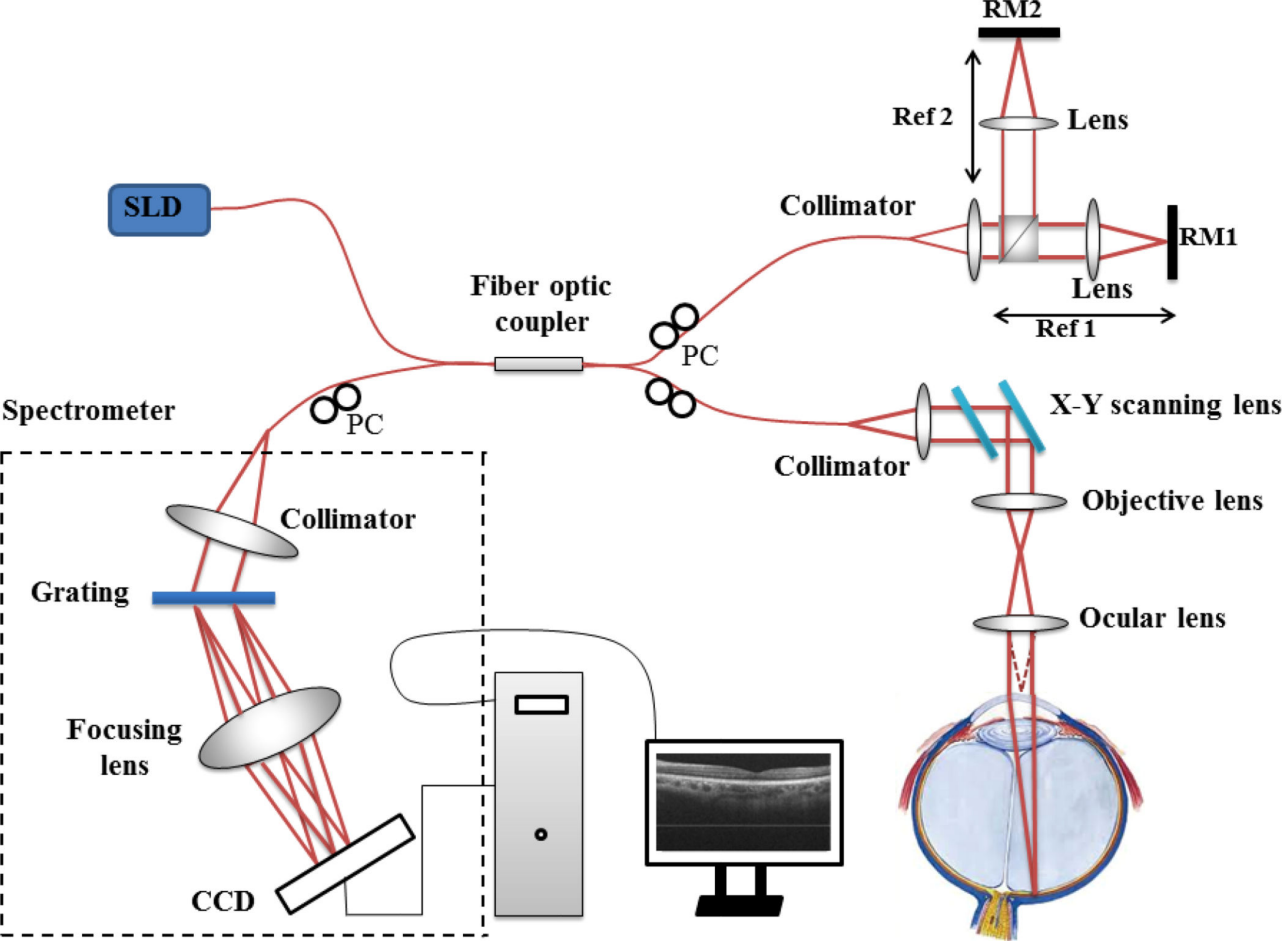
Schematic of the ultrahigh-speed SDOCT system setup. SLD: 1050 nm superluminescent diode; PC: polarization control.

The schematic of the 1050 nm SD-OCT system is illustrated in Fig. 1. The setup for retinal imaging is similar to that described in a previous study.^21,22^ Briefly, the system employs a 1050 nm ASE module (Amonics, ALS-1050-20) as the light source, with an output power of ∼20mW. The light module emits light with 1050 nm central wavelength and a bandwidth of 50 nm, delivering a measured axial resolution of ∼ 10 µm in air (∼7µm in tissue). The output from the light source is split by a 10:90 fiber coupler into the sample (10%) and reference arms (90%). In the sample arm, light from the single-mode fiber is collimated with a fiber collimator to produce parallel illumination on the X-Y 2D scanning system and then on the objective lens and ocular lens. Combined with the ocular lens, the focus spot of the probe beam at retinal surface is estimated at ∼20 µm, which determines the system lateral resolution. In the reference arm, a beam splitter is used to split light into two separate reference beams that are relayed onto two separate reference mirrors, RM1 and RM2. This setup enables simultaneous measurement of IOL and CT in one scan (see Sec. 3). The RM1 is aligned for targeting the cornea, whereas the RM2 is designed for 3D imaging of choroid and retina. Both lights reflected from RM1 and RM2 are reflected back into the system and mixed with the light backscattered from the sample. The mixed lights are then detected by an ultrafast spectrometer, consisting of a fiber collimator, a diffraction grating (1200 lines/mm), an achromatic lens, and a high speed line-scan InGaAs camera (120 kHz, 2048 × 10 µm, United Technologies Aerospace Systems, New Jersey, USA). The signal captured by the line-scan camera is transferred to the computer through a camera link for implementing fast Fourier transform to gain the depth-scan (A-scan) of OCT imaging. The spectral resolution of the spectrometer is ∼0.07nm, leading to ∼ 6.1 mm extended imaging depth for the OCT prototype system. Due to the high-speed (120 kHz) camera employed, 3D data volume can be captured in ∼ 1 s while the axial resolution is not sacrificed, which is helpful for the accurate measurement of CT of human eyes. With the RM2 targeting at the retina, the system sensitivity at 0.5 mm depth was measured at ∼100 dB, with a 6 dB sensitivity rolling-off at ∼3 mm depth position and a 12 dB at 6 mm depth position.

### 2.3. Imaging the posterior segment and measuring IOL in one scan

The IOL of the normal adult is typically between ∼ 23 and ∼ 24 mm, which is much longer than the depth range of the SD-OCT system (∼ 6.1 mm), making it impossible to measure it while providing the CT imaging at the same time by the use of conventional SD-OCT setup. To mitigate this problem, we propose to employ two separate reference arms (see Fig. 1), enabling independent access to two separate ranging distances, each targeting at the cornea and retina, respectively. Both the RM1 and RM2 are mounted on precision-micrometer translation stages (travel distance 25.000 ± 0.001 mm). To achieve simultaneous access to the cornea and the retina during one scan, a calibration procedure of the relative position between RM1 and RM2 is necessary for the precise measurement of IOL.

**Fig. 2:**
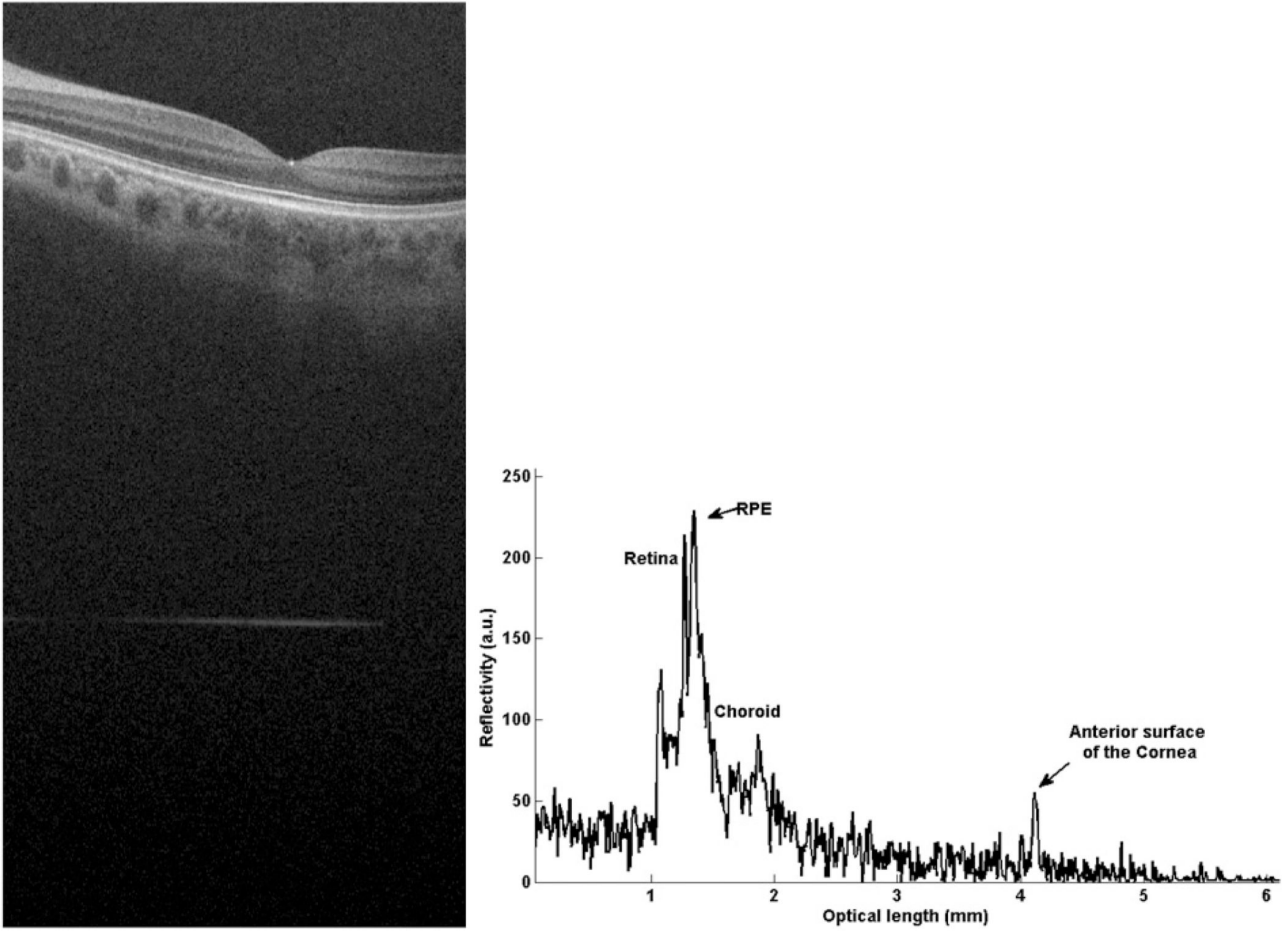
Composite A-scan of low myopic human eye *in vivo*. The retina is located close to the origin of the graph and the front surface of the cornea further away. (a) gives the cross section OCT image, which contains the retinal image and corneal signal. (b) is the A-line information for the IOL measurement. For this subject, the AL calculated by the formula is (35 – 2.236)/1.3375 = 24.496 mm. For comparison, length measurement data of myopic eyes with IOL master also has been conducted. The comparisons between our SD-OCT system and IOL master results are given in Table 1.

First of all, with RM1 blocked, the system was optimized for retinal imaging. To perform the calibration procedure, a mirror was used to simulate a sample in the sample arm. In this case, while the RM2 was fixed, the RM1 was moved until the peaks of the low coherence interferogram from the each reference beams were overlapped, meaning that both RM1 and RM2 give the same optical path length in the reference arm. We defined this position of RM1 as the arbitrary zero position. And then, the optical path length defined by the RM1 was reduced by precisely translating the RM1 by Δ*L*_ref_ distance to target the cornea. This Δ*L*_ref_ defines the reference-mirror offset, i.e., the optical path length difference between RM1 and RM2. In this study, Δ*L*_ref_ = 35 mm. Taking into account a group refractive index (*n*) of 1.3375 for the eye, the 35 mm effective path length in air is approximately 2 mm longer than average IOL of normal subjects. During imaging, the RM1 defines the position of the anterior surface of cornea in the OCT image generated by the system. Typically, the position of the anterior surface of cornea appeared in the OCT image is about 4 mm away from the zero delay line, leaving about 4 mm space in the output plane for retinal imaging. Figure 2(a) gives an example of a representative OCT cross section image acquired from one of the subjects, where the line appearing in the bottom (arrow pointed) is the signal due to the anterior surface of cornea, while the cross-sectional retinal image appears between this line and the zero-delay line.

To calculate the IOL, the A-scan passing through the fovea aligned with the optical axis was extracted [see Fig. 2(b)], and the distance *d* between the fovea and the cornea surface was evaluated. The IOL was then determined by Δ*L*_ref_ subtracted by that distance, and then scaled by the group refractive index, i.e., IOL = [Δ*L*_ref_− *d*]/*n*. For this example of Fig. 2(a), *d* measures 2.236 mm; thus, the IOL is calculated by the formula (35 – 2.236)/1.3375 = 24.496 mm.

The setup was cross-validated by separate measurements employing an IOL Master. Five subjects participated. Measurements in each subject were performed by using the OCT system and the IOL Master, separately. Each reading was obtained by averaging five repeated measurements. Note that the subject’s head was repositioned between each measurement. The comparison between the SD-OCT system and IOL master data are given in Table 1, demonstrating an excellent agreement, with deviations less than the axial resolution of the OCT system as expected.

### 2.4. Geographic measurement of CT

**Fig. 3:**
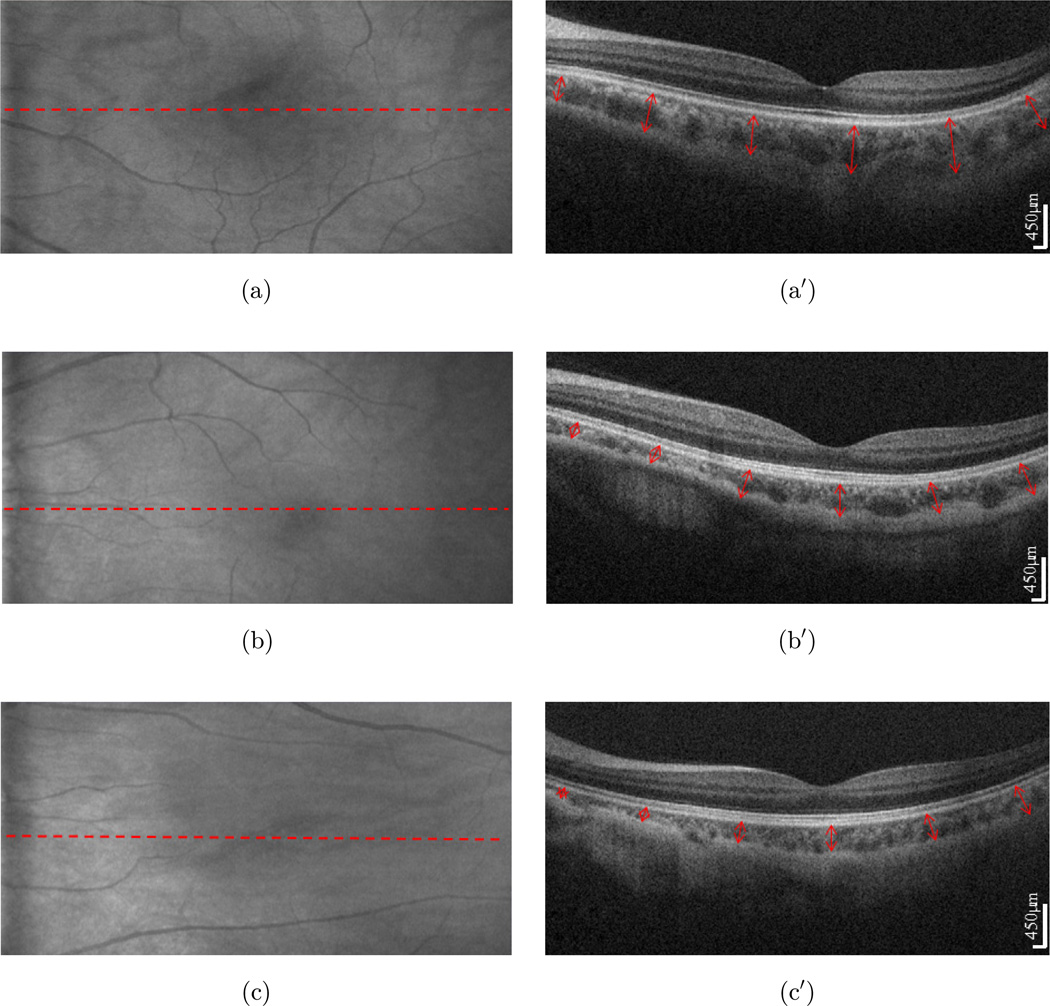
The CT of myopic eyes. (a), (b) and (c) is the projection of the low (−1D), moderate (−5D) and high myopic (−8D) eye, respectively. The choroid is shown on the cross section SDOCT scan (a′) (b′) and (c′). The CT is measured vertically from the outer border of the RPE to the outer border of the choroid (shown with red arrow).

To measure geographic CT, 3D OCT images were acquired from each subject. For OCT scanning, 512 A-lines formed one B-scan and 256 B-scans formed one 3D image, covering an area of ∼ 10 × 5 mm on the retina approximately centered at fovea. Using this scanning protocol with the A-line rate of 120 kHz, the system imaging speed was 220 frames per second. With this speed, it took only ∼ 1.2 s to complete one 3D scan. Figure 3 shows typical SD-OCT images acquired from the low, moderate and high myopic eyes, respectively, where the left images resulted from maximum projection (i.e., OCT fundus image) and the right images were the cross section OCT images at the position marked by the red lines in the left, upon which the CT was measured.

Following the convention in the literature, the CT is defined as the distance from the outer surface of the hyper-reflective line, the RPE layer, to the hyper-reflective line of the inner sclera border.^25^ In our measurement, the CT was measured in the direction normal to the curve defined by the RPE layer, as seen by the red arrows in the left images of Figs. 3(a′)–3(c′). Simply from the cross section OCT images, the variation of CT among low, moderate and high myopia is visible. The CT is thicker in low myopia than in other conditions and the thinnest CT appears in the high myopic case.

**Fig. 4:**
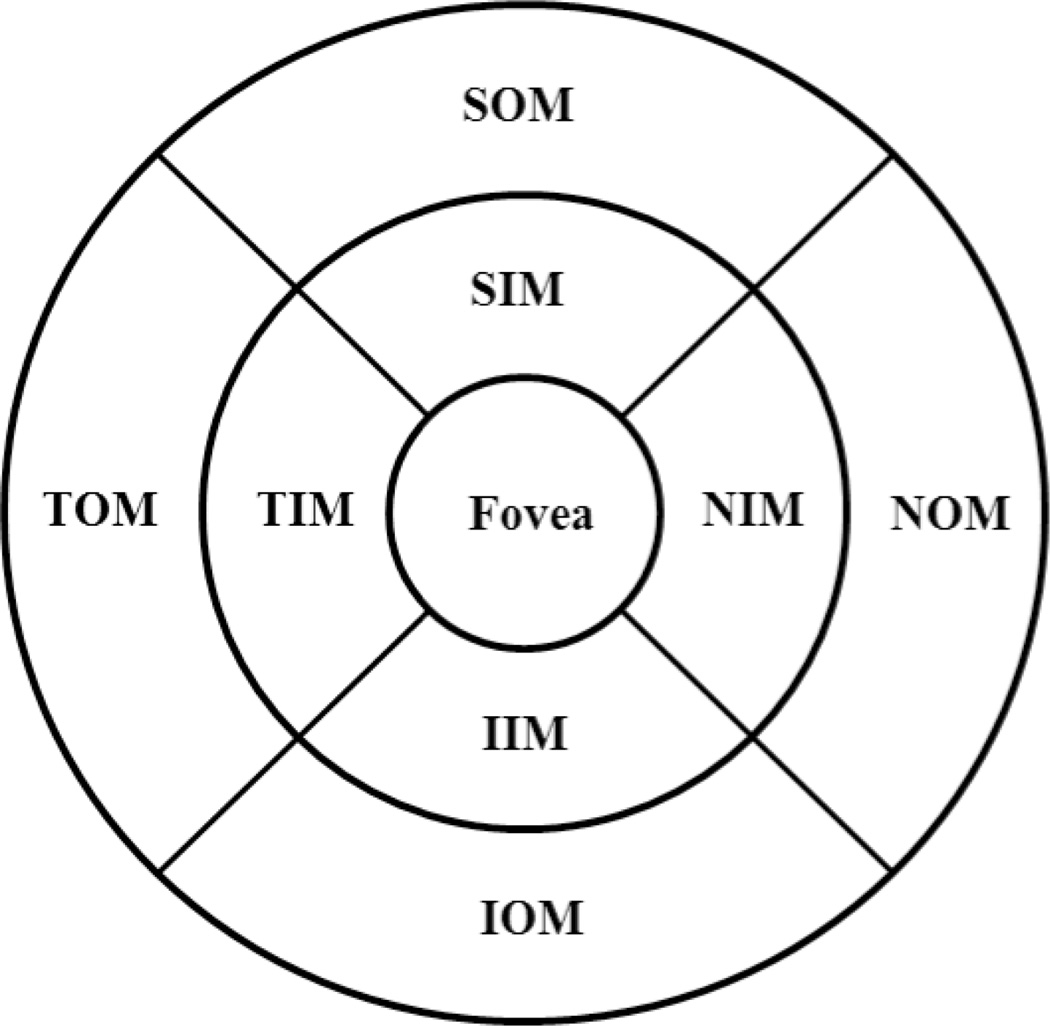
Segmentation of superior, inferior, nasal, and temporal quadrants. Fovea: the central fovea, NIM: nasal inner macula, SIM: superior inner macula, TIM: temporal inner macula, IIM: inferior inner macula, NOM: nasal outer macula, SOM: superior outer macula, TOM: temporal outer macula, IOM: inferior outer macula.

To obtain geographic CT map, semi-automated retinal layer segmentation software developed in house^26^ was used to segment the choroidal layer from the OCT cross sectional structural images. Before segmentation, any possible motion artifacts were removed from all the OCT cross section images by the use of cross-correlation approach.^27–29^ After segmentation, the 3D segmented image, representing CT, was divided into superior, inferior, nasal, and temporal quadrants, as depicted in Fig. 4, and the average thickness of choroid in each quadrant was calculated. The three defined rings in Fig. 4 have the diameters of 1, 5, and 10 mm respectively. Individual sectors were referred to as the central fovea (Fovea), nasal inner macula (NIM), superior inner macula (SIM), temporal inner macula (TIM), inferior inner macula (IIM), nasal outer macula (NOM), superior outer macula (SOM), temporal outer macula (TOM), and inferior outer macula (IOM), respectively. The average thickness was then calculated on each separate sector. In another measurement in order to investigate more localized CT distribution in the nasal-temporal direction, the number of concentric rings is increased in the map of Fig. 4 so that the distance between the adjacent rings is 1 mm. The average CT is then calculated in each narrower ring-sector.

### 2.5. Statistical analysis

Data are presented as mean ± STD. The differences of CT and IOL among low, moderate and high myopic eyes were analyzed using Student’s *t*-test by determining the *p*-value, where it is assumed that the sample populations are normally distributed. The criterion for a statistical significance is *p* < 0.05.

## 3. Results and Discussion

**Fig. 5:**
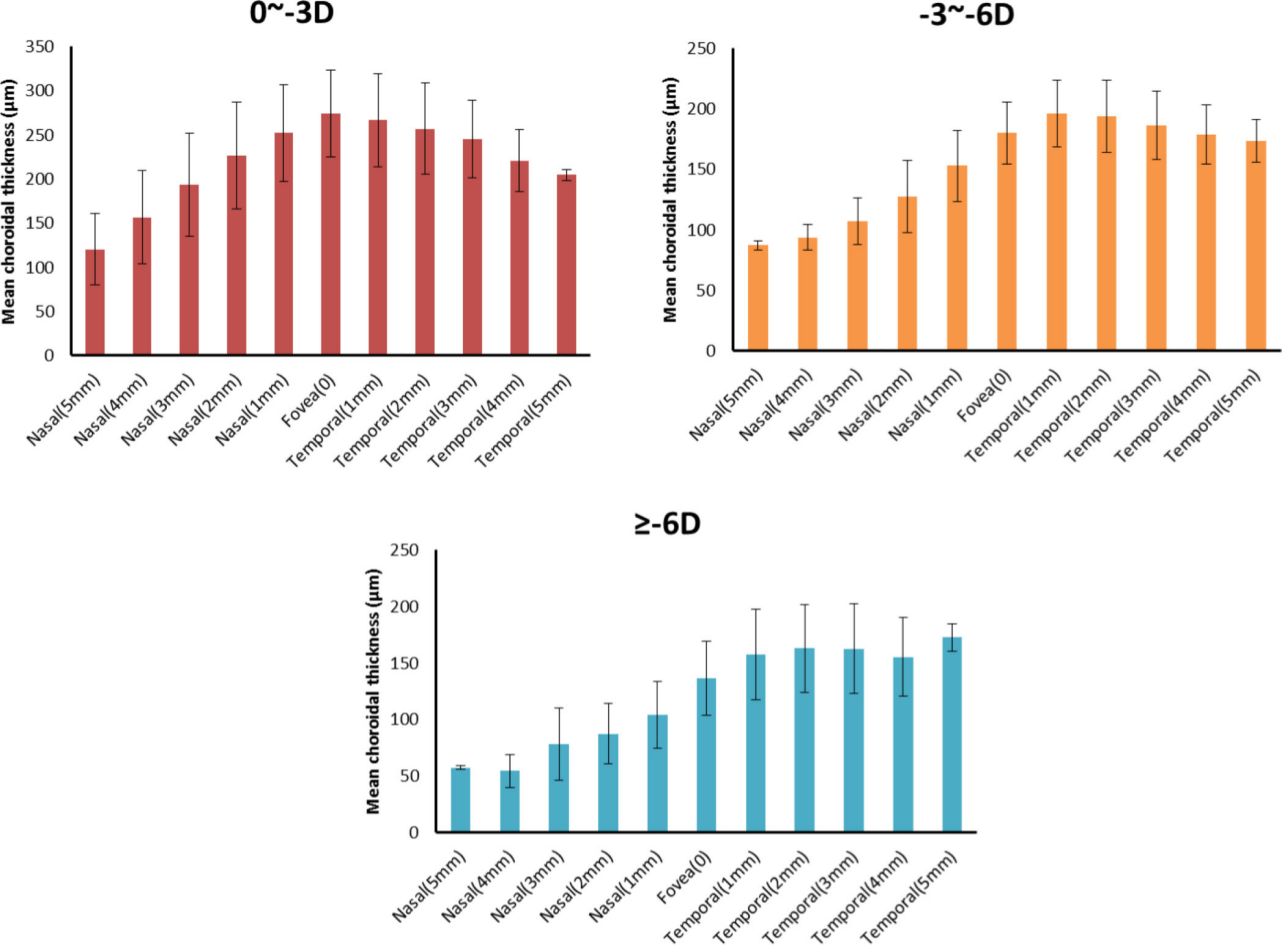
The CT distributions from nasal to fovea to temporal with an interval of 1 mm of low, moderate, and high myopia.

**Fig. 6:**
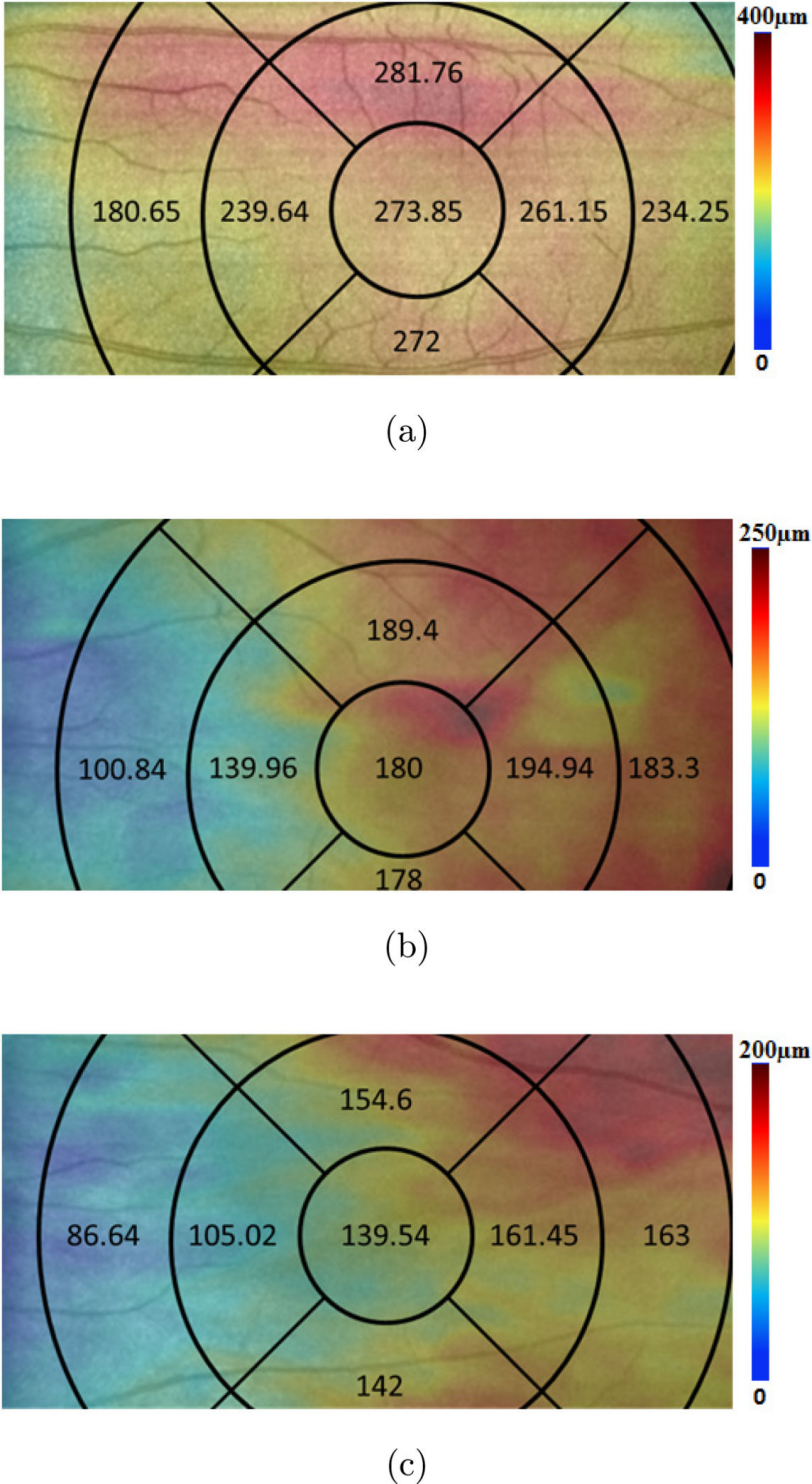
The mean CT distribution in different superior, inferior, nasal, and temporal quadrants under conditions of low (a), moderate, (b) and high myopia (c). Data are presented as the mean value.

Segmentation was performed twice on each subject by two operators, and results were excluded if the discrepancy between the analyses was more than 15%. The CT measurements were performed after the layer segmentation of the retinal structure images. The mean values and standard errors were generated for analysis. Figure 5 depicts the CT distribution from the nasal side to the fovea to the temporal side with an interval of 1 mm (the ring-disk thickness in Fig. 4 is 1 mm) over low, moderate, and high myopia. In low myopic eye, the thickest CT in nasal-temporal direction is under the fovea (subfoveal) while the thinnest is in the nasal region. However, for eyes with moderate and high myopia, the thickest measures were found in the temporal region. The thinnest location remained in the nasal direction. The differences in CT distribution tendency across the low, moderate, and high myopias is interesting. Table 2 gives the mean value and standard error of CT in different positions based on the quadrants shown in Fig. 4. The mean CT distribution of 3D data volume were calculated (Fig. 6) at different quadrant sectors (the ring-disk thickness is same with Fig. 4) and colored mapped onto the OCT fundus image for low, moderate, and high myopic conditions, respectively.

**Fig. 7:**
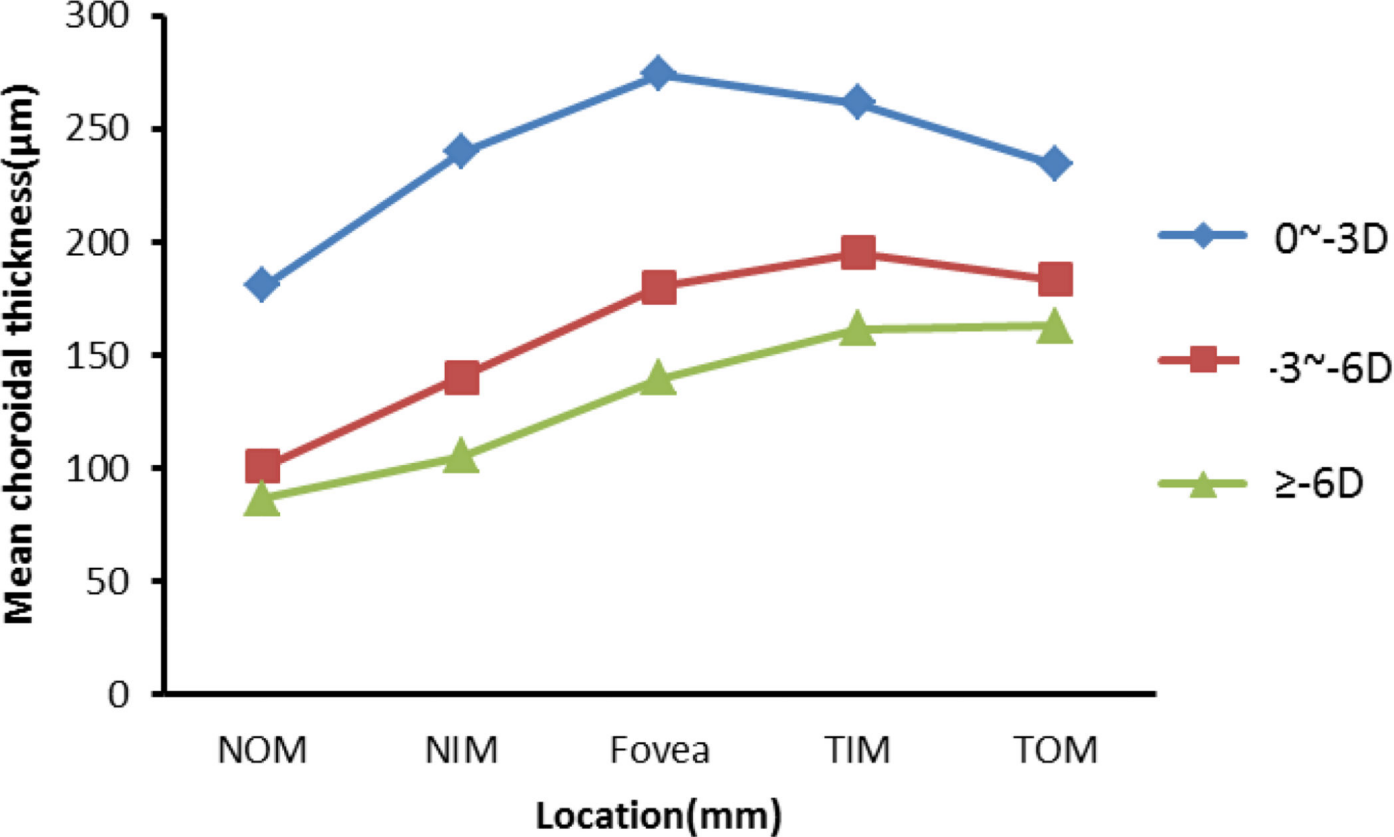
The distribution of CT in fovea, temporal, and nasal of low, moderate, and high myopia. The thickest CT lies under the fovea in low myopia, and is thinnest under the nasal region. However, in moderate and high myopia the thickest CT is in the temporal zone and thinnest under the nasal.

The results indicate that for eyes with low myopia the thickest position (273.85 ± 49.01 µm) was found under the fovea as is the case with healthy people.^30^ Outside the fovea, the mean CT decreased rapidly in the nasal direction, reaching a minimum of 180.65 ± 58.25 µm at a distance of 5 mm from the fovea. In the temporal direction, the thickness also decreased, but less dramatically. The mean thickness in the temporal region at 5 mm from the fovea was 234.25 ± 42.27 µm. In contrast to the low myopic results, the thickest region for the moderate and high myopias was not under the fovea, but in the temporal region (Fig. 6). These results are similar to those reported by Takamitsu *et al*.^15^ However, for high myopic eyes, CT varied from a maximum of 163 ± 34.89 µm in the temporal region to 86.64 ± 42.6 µm in the nasal region. There is no existing literature on moderate myopia; however its study may be important for understanding the progress of myopia and for developing interventions. Our findings indicate that CT in moderate myopia tends toward measurements found in high myopia, as the temporal region is thickest measuring at 194.94 ± 27.28 µm while the nasal region is thinnest at 100.84 ± 16.75 µm. It would appear that in conditions of moderate and high myopia the thickest portion of the choroid moves from the fovea toward the temporal region. This change was less pronounced in moderate myopia, as the thickest part of the choroid is closer to the fovea than that in high myopia. Figure 7 gives the distribution tendency of CT in different locations for comparison. From the tendency distribution, the thickest CT location varies according to the severity of myopia.

**Fig. 8:**
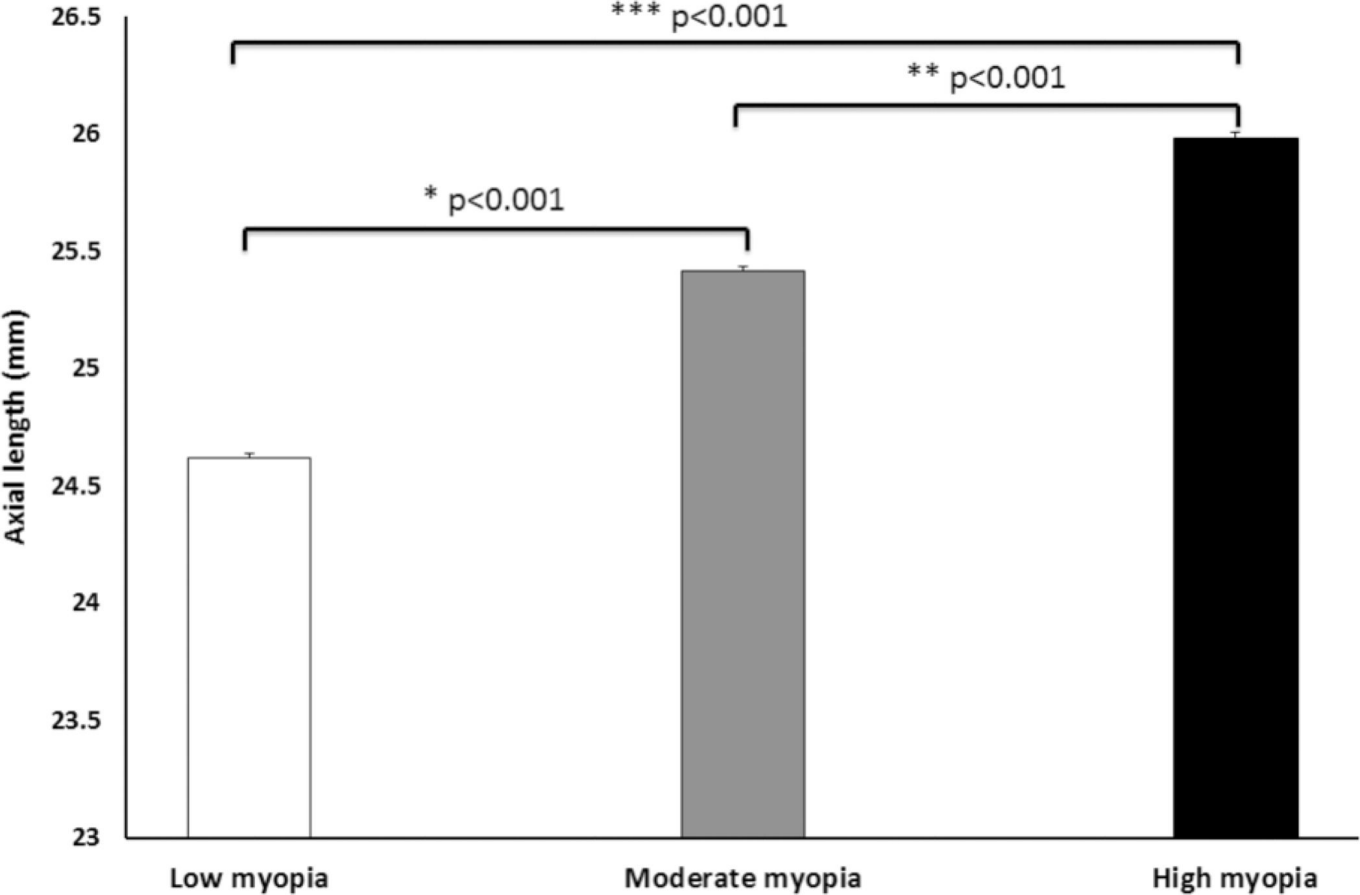
The IOL of subjects under conditions of low, moderate, and high myopia. Data are presented as mean ± STD. *Note*: *p < 0.001 indicates significant difference between low and moderate myopia; **p < 0.001 indicates significant difference between moderate and high myopia; ***p < 0.001 indicates significant difference between low and high myopia.

Besides the changes of CT in myopia, the IOL is also affected due to axial elongation associated with myopia. The IOLs for the low, moderate and high myopic eyes are shown in Fig. 8. The differences are statistically significant. Low myopic eyes showed a shorter mean IOL (24.619 ± 0.016 mm), while high myopic eyes were longest (25.983 ± 0.021 mm, *** *p* < 0.001). The moderate myopic eyes have longer IOLs (25.413 ± 0.022 mm, * *p* < 0.001) than the low myopic eyes. In high myopia, the excessive axial elongation of the eye may lead to macula holes or tears in the retina, causing retinal detachment. People with high myopia need comprehensive dilated eye exams more often and early detection and timely treatment could help prevent vision loss.

**Fig. 9:**
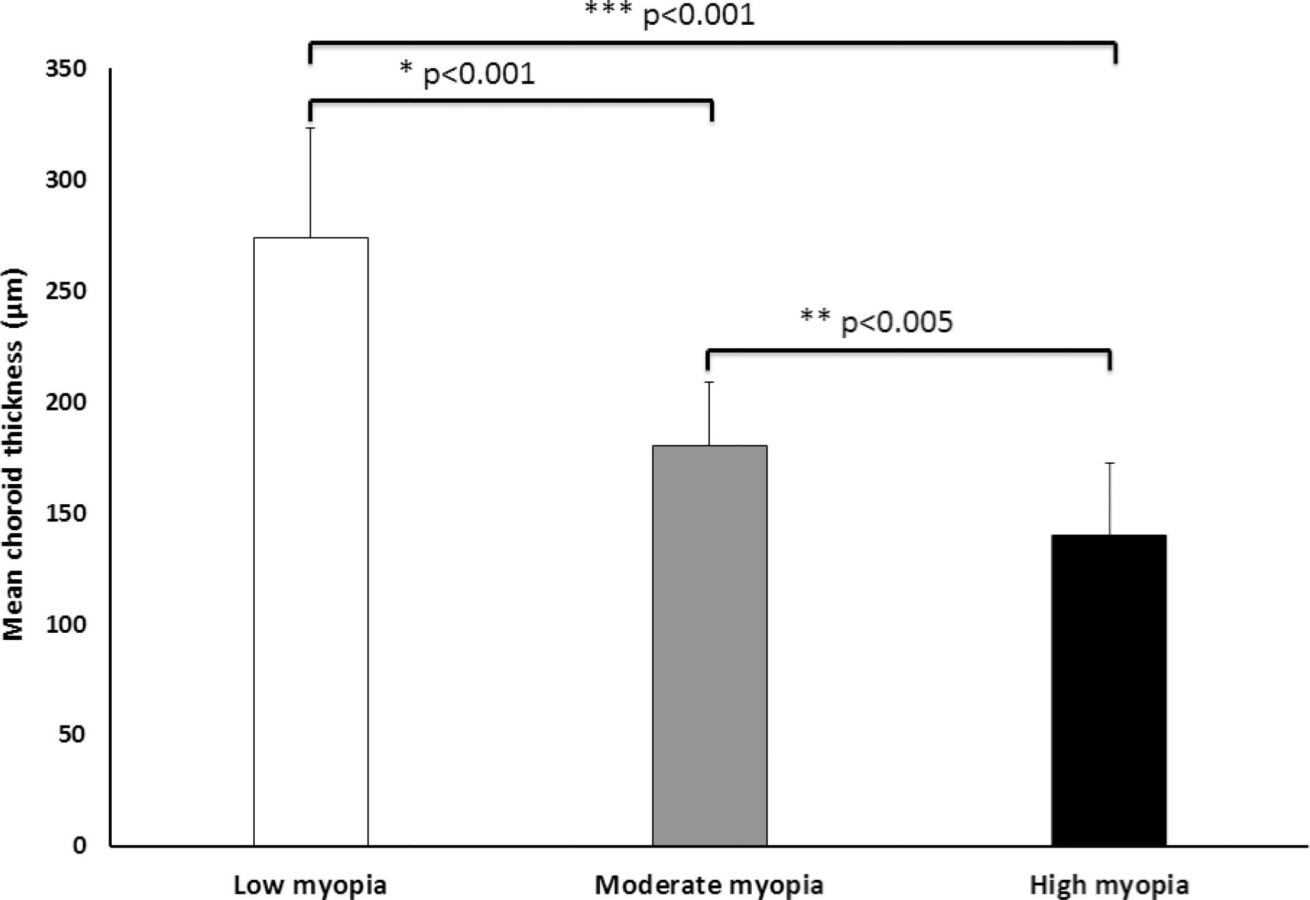
CT changes under the fovea in low, moderate and high myopia. Data are presented as mean ± STD. *Note*: *p < 0.001 indicates significant difference between low and moderate myopia; **p < 0.005 indicates significant difference between moderate and high myopia; ***p < 0.001 indicates significant difference between low and high myopia.

Another interesting result is that CT decreased rapidly from low myopia to moderate myopia and the difference is highly statistically significant (*p < 0.001); however from moderate to high myopia, although significant (**p < 0.005), the decrease of thickness was relatively small. For this reason, we further studied the relationship between CT and refractive error (diopters). The reduction in CT per diopter from low to moderate myopia was 31.28 µm/D, while the reduction in CT from moderate to high myopia was much smaller at 13.49 µm/D. This result was calculated based on the thickness under the fovea. Figure 9 summarizes the distribution of CT under the fovea in each myopia conditions. There are significantly statistical differences found between each pair of conditions (between low and moderate myopia, *p < 0.001; between moderate and high myopia, **p < 0.005; between low and high myopia, ***p < 0.001). Corresponding to the IOL variation, the myopic eyes have longer IOL, but thinner CT than normal eyes. As the degree of myopia increases, axial length increases gradually and the CT is progressively thinner. This may be helpful for the study of the role of the choroid in the development of myopia. The choroid provides oxygen and nourishment to the cells of the RPE and outer layers of the retina. In addition, it also contributes to the blood supply of the prelaminar and anterior portions of the optic nerve, as well as the macula, which is crucial for central vision.^31^ These findings may be clinically significant for diseases affecting choroidal blood supply. Low myopic eyes are quite similar to normal eyes where the CT thickness is little changed from normal and the supply from choroidal blood vessels appears close to normal. Compared to the low myopic eye, moderate myopic eyeball have a reduced choroidal blood supply and a large reduction in thickness. High myopic eyeballs are even further elongated, and CT is further reduced. However, compared to the change between low and moderate myopia, this reduction is relatively small.

## 4. Conclusion

We investigated the changes in CT and IOL among low, moderate and high myopic eyes using a highspeed two-separated reference arms SD-OCT system. To the best of our knowledge, this is the first study to report the measurements of CT and IOL, simultaneously, among the eyes with low, moderate and high myopic conditions. We found that overall CT is reduced as a function of refractive power in myopia, i.e., low myopic eyes possess the thickest CT, while high myopic eyes have the thinnest CT but the longest IOL. Low myopic eyes have shorter IOL than high myopic eyes do. The thickest region of the choroid in moderate and high myopia migrates progressively away the fovea compared to low myopia. CT decreases most rapidly from low to moderate myopia and from moderate to high myopia, this decrease in thickness is relatively small. These findings will be helpful for further investigations of the progress and mechanism of myopia.

This study is subject to the following limitations; all segmentation measurements on the SD-OCT images were performed semi-automatically, and it was a retrospective study with a small number of subjects. This study also lacked long-term follow-up, and the changes in CT may not occur at the same rate between patients. More systematic studies are warranted to mitigate these limitations.

## Figures and Tables

**Table 1:** Measurement of the myopic eye length *in vivo* with the SD-OCT system and with IOL master.

	SD-OCT system (mm)		IOL Master (mm)		Difference (mm)	
Subject #	Right eye	Left eye	Right eye	Left eye	Right eye	Left eye
Subject 1	24.406	24.257	24.41	24.26	−0.004	−0.003
Subject 2	24.181	24.409	24.18	24.41	0.001	−0.001
Subject 3	25.129	25.640	25.12	25.60	0.009	0.04
Subject 4	24.456	24.603	24.46	24.60	−0.004	0.003
Subject 5	27.831	27.847	27.82	27.85	0.011	−0.003

**Table 2:** CT in different positions under conditions of low, moderate and high myopia. Data are presented as mean ± STD.

D*	NOM (µm)	NIM (µm)	Fovea (µm)	TIM (µm)	TOM (µm)
0∼ −3	180.65 ± 58.25	239.64 ± 57.93	273.85 ± 49.01	261.15 ± 51.62	234.25 ± 42.27
−3 ∼ −6	100.84 ± 16.75	139.96 ± 31.53	180 ± 28.25	194.94 ± 27.78	183.3 ± 24.38
> = −6	86.64 ± 42.6	105.02 ± 34.14	139.54 ± 32.68	161.45 ± 25.23	163 ± 34.89

*Note:* *Diopters.
